# Biochar and gypsum amendment of agro-industrial waste for enhanced black soldier fly larval biomass and quality frass fertilizer

**DOI:** 10.1371/journal.pone.0238154

**Published:** 2020-08-27

**Authors:** Dennis Beesigamukama, Benson Mochoge, Nicholas K. Korir, Komi K. M. Fiaboe, Dorothy Nakimbugwe, Fathiya M. Khamis, Thomas Dubois, Sevgan Subramanian, Musyoka M. Wangu, Sunday Ekesi, Chrysantus M. Tanga

**Affiliations:** 1 International Centre of Insect Physiology and Ecology, Nairobi, Kenya; 2 Department of Agricultural Science and Technology, Kenyatta University, Nairobi, Kenya; 3 Department of Crop Production and Management, Busitema University, Kampala, Uganda; 4 International Institute of Tropical Agriculture, Yaoundé, Cameroon; 5 Department of Food Technology and Nutrition, School of Food Technology, Nutrition & Bioengineering, Makerere University, Kampala, Uganda; Kyonggi University, REPUBLIC OF KOREA

## Abstract

Black soldier fly (BSF) (*Hermetia illucens* L.) is one of the most efficient bio-waste recyclers. Although, waste substrate amendments with biochar or gypsum during composting process are known to enhance nutrient retention, their impact on agro-industrial waste have not been documented. Hence, this study focuses on a comparative effect of agro-industrial waste amended with biochar and gypsum on BSF larval performance, waste degradation, and nitrogen (N) and potassium retention in frass fertilizer. Brewery spent grain was amended with biochar or gypsum at 0, 5, 10, 15 and 20% to determine the most effective rates of inclusion. Amending feedstock with 20% biochar significantly increased wet (89%) and dried (86%) larval yields than the control (unamended feedstock). However, amendment with 15% gypsum caused decrease in wet (34%) and dried (30%) larval yields but conserved the highest amount of N in frass. Furthermore, the inclusion of 20% biochar recorded the highest frass fertilizer yield and gave a 21% increase in N retention in frass fertilizer, while biomass conversion rate was increased by 195% compared to the control. Feedstock amendment with 5% biochar had the highest waste degradation efficiency. Potassium content in frass fertilizer was also significantly enhanced with biochar amendment. At maturity, frass compost with more than 10% inclusion rate of biochar had the highest cabbage seed germination indices (>100%). The findings of this study revealed that initial composting of biochar amended feedstocks using BSF larvae can significantly shorten compost maturity time to 5 weeks with enhanced nutrient recycling compared to the conventional composting methods.

## Introduction

In recent years, the efforts to recycle organic wastes using black soldier fly (BSF) (*Hermetia illucens* L.) larvae into high-quality sustainable protein to substitute fishmeal in animal feeds and frass fertilizer for organic farming have gained momentum worldwide [1–4]. Black soldier fly larvae are voracious consumers of a wide range of organic waste including crop [5–7], animal [8,9] and human wastes [10–13]. However, the performance and nutritional quality of the larvae largely depend on the quality of the substrates used during rearing [1,14]. Most organic wastes in sub-Saharan Africa have been reported to be of low nutrient quality [15–17], which might probably be attributed to the excessive loss of nitrogen (N) through ammonia volatilization [11]. It is important to note that ammonia is a greenhouse gas and its emissions to the atmosphere are detrimental to both human health and environment in terms of its harmful effects on animals, plants, and soil ecosystems [18,19]. The increasing loss of N during the composting process leads to frass that is extremely poor in quality and therefore not fit for use as organic fertilizer.

It has been demonstrated by Oonincx et al. [9] that approximately 23–78% of substrate N from chicken, pig and cow manure is lost during composting using BSF larvae. Furthermore, BSF larvae have been shown to thrive best on waste substrates with neutral to alkaline pH [20] although pH values above 7.5 have been reported to favour nitrogen loss through ammonia volatilization [21,22]. This is because an increase in pH has been shown to increase the dissociation of ammonium (NH_4_^+^) to ammonia gas (NH_3_), thus shifting the equilibrium to NH_3_ which eventually evaporates [23]. Therefore, the amendment of substrates to produce high quality frass for better quality organic fertilizer production would constitute an additional value-added product from the rearing system, which will directly accrue revenue to smallholder farmers engaged in insect-based enterprises. On the other hand, the organic fertilizer generated from the BSF rearing process would play an essential role in soil fertility replenishment and reduce the constant reliance on mineral fertilizer inputs for crop production.

Studies have shown that one of the major ways of conserving N in composting substrates is the control of ammonia volatilization through the addition of substances that can stabilize ammonium ions either through ammonium precipitation, adsorption, or both [23]. Ammonium precipitation usually happens when salts are added to ammonium ions which chemically combine to form stable compounds such as ammonium sulphate and ammonium phosphate [24,25]. Also, the addition of 17% gypsum to composting substrate has been found to improve phosphorus (P) and ash contents, while reducing N loss by 94% and pH from 7.6 to 7.3 [26]. Gypsum is well known for reducing N diffusion and converting ammonium carbonate to ammonium sulphate as described above [23]. The use of 20% biochar and 20% flue gas desulfurization gypsum during chicken litter composting was found to reduce ammonia loss by 68 and 24%, respectively, thus significantly increasing the nutrient levels of compost [27]. Other studies have also demonstrated that composting of kitchen waste with 10% phospho-gypsum can reduce ammonia volatilization by 24% and conserve total N by 17% with improved nutrient content of the compost [28].

The second strategy for conserving N involves ammonium adsorption, which can be achieved through the adhesion of ammonium ions on negatively charged materials such as biochar, peat, zeolite, and clay [23,29]. This action reduces ammonium ion concentration in liquid phase and minimizes nitrogen loss as ammonia gas and other forms [30]. In addition, high surface charged materials such as biochar can also prevent further loss of ammonia by reducing the activities of nitrifying bacteria [31,32]. Furthermore, more nitrogen loss can be controlled through absorption of ammonium ions into the pore spaces of biochar [29]. Through such mechanisms described above, some studies have demonstrated that biochar inclusion during organic waste composting can reduce ammonia volatilization by up to 60% [33], thus increasing total N content in compost. Apart from improving the agronomic value of compost, biochar amendment has been shown to safeguard the environment by the reduction of greenhouse gas emissions [34–36].

Although biochar inclusion rates of 5–10% have been reported to be effective in reducing ammonia losses, higher rates above 20% have been found to have a negative effect on microbial activity, which can slowdown the composting process [37]. Unlike the conventional composting process which is mediated by microorganisms only [38], the use of BSF to compost organic waste amended with biochar and gypsum is unknown. The effective rates of inclusion of these amendments for optimal growth of BSF larvae and yield have not been documented. Also, the effects of substrate amendments with biochar and gypsum on the pH, moisture content, electrical conductivity, temperature and frass quality have not been reported. Furthermore, information on nutrient release and the time required to achieve frass compost maturity needs to be documented. The present studies, therefore, sought to evaluate the potential impacts of rearing substrate amendment using biochar and gypsum on BSF larval performance and quality of the frass fertilizer generated.

## Materials and methods

### Black soldier fly colony establishment and maintenance

The stock colony of BSF was established at the Animal Rearing and Quarantine Unit (ARQU) of the International Centre of Insect Physiology and Ecology (*icipe*), following the methodology described by Chia et al. [39]. The colony was initiated from eggs collected from wild adult BSF populations at Kasarani area, Nairobi County, Kenya (S 01° 13' 14.6''; E 036° 53' 44.5'', 1,612 m above sea level). A combination of 2-week-old fermented chicken and rabbit manure mixed with fruits and household food wastes placed in buckets was used as baiting material for adult flies. The buckets were checked regularly (2–3 times per week) for deposited egg clusters in the cardboard flutes. Thereafter, the egg clusters were transferred into new containers with substrates comprised of kitchen waste, brewers’ spent grain and market waste (fruits and vegetables) for rearing the newly hatched neonates. Conditions in the rearing room were maintained at 28 ± 2 °C, 60–70% relative humidity and a photoperiod of L12:D12. Portable digital thermo-hygrometers were placed inside each of the rearing rooms to monitor temperature and relative humidity. After 12–14 days of rearing, the prepupal stages were harvested from the rearing trays and placed into 2-litre transparent rectangular plastic containers (Kenpoly, Nairobi, Kenya) containing 60% of moist sawdust to prevent desiccation. The prepupae grew to pupal stages in the same containers. After 8–10 days, adult flies emerged and were transferred into large outdoor cages (1 m × 1 m × 1.8 m) holding approximately 2,000–2,500 adult flies per cage. While in the cages, the flies were provided with 60% sugar solution soaked on cotton wool.

### Experimental diets

Brewery spent grains (BSGs), suitable for BSF rearing [8,40] was sourced from East African Breweries Ltd, Nairobi, Kenya. Biochar made from rice husks at pyrolysis temperatures of 350 °C was sourced from SAFI Organics Limited, located in Mwea, Kirinyaga County, Kenya. Gypsum (calcium sulphate) was sourced from the Kenya Farmers’ Association stores, Nairobi. Detailed analyses of the nutrient levels [carbon, N, P, potassium (K), calcium (Ca), and magnesium (Mg)], moisture content, pH, electrical conductivity (EC) of BSGs, biochar and gypsum were conducted using recommended laboratory methods [41]. The results are presented in Table 1.

**Table 1:** Selected physical and chemical characteristics of brewery spent grain, biochar and gypsum used in the experiments.

Substrate	Moisture content (%)	pH (1:10 water)	EC (mS cm^-1^)	TOC	Total N	Total P	Total K	Total Ca	Total Mg	C/N ratio
				…………………. (%) …………………….						
BSGs	71.4	3.5	0.86	33.9	2.65	0.54	0.38	0.48	0.20	12.7
Biochar	8.9	7.7	0.51	22.6	0.46	0.014	2.67	0.14	0.86	49.1
Gypsum	0.62	8.0	4.24	NA	0.00	0.003	0.05	25.1	0.43	NA

EC = electrical conductivity, TOC = total organic carbon, N = nitrogen, P = phosphorus, K = potassium, Ca = calcium, Mg = magnesium, C/N = carbon to nitrogen and NA = not applicable, BSGs = brewery spent grains

Based on the analyses described above, the BSGs were amended with biochar at different inclusion rates of 5, 10, 15 and 20% dry weight (weight /weight) to obtain four treatment regimes, denoted as 5Biochar, 10Biochar, 15Biochar and 20Biochar, respectively. The BSGs were also amended with gypsum at three different inclusion rates of 5, 10 and 15% dry weight (weight /weight), represented as 5Gypsum, 10Gypsum and 15Gypsum, respectively. The control treatment was neither amended with biochar nor gypsum.

### Black soldier fly larval growth and yield on various substrates

The rearing facility of BSF larvae was equipped with a wooden stand (180 cm high × 66 cm wide × 420 cm long). The wooden stand had three shelves separated from each other by a 30 cm space, where the metallic trays used in rearing the BSF larvae were fitted. Metallic trays used during the experiment measured 76 cm long, 27.5 cm wide and 10 cm deep. The bottom of each tray measured 52 cm in length by 27.5 cm width, which allowed for both edges of the tray to be inclined at an angle of 35°. Both ends of each tray were provided with a collar of 5 cm long to allow it sit smoothly on the edge of each trough. In the experimental room, 8 trays were fitted per shelf of the wooden stand, totalling to 24 trays arranged in a complete randomized design.

At the start of the experiment, eggs of BSF were hatched and larvae reared on each of the eight substrate treatments described above. After 5 days, 2,000 neonates from each treatment were collected and transferred directly to their respective treated substrates (7.10 kg) in metallic trays. The recommended feeding rate per larva per day was 125 mg (dry weight) [42]. Each rearing substrate was hydrated to approximately 70.0 ± 2% moisture by weight according to the protocol described by Cammack and Tomberlin [43], and confirmed using a moisture sensor with two 12-cm-long probes (HydroSense CS620, Campbell Scientific, Logan, USA). Three replicates were conducted for each experimental substrate fed *ad libitum* until the last larval stage (i.e. 5^th^ instar larvae) was completed. The experiment was conducted in two sets.

The experimental room was heated using fast moving dry hot air at 28.0 ± 2 °C (using Xpelair heater, Wall Fan Heater, UK) with thermoregulators. Portable digital thermo-hygrometers were placed inside each of the rearing rooms to monitor temperature and relative humidity. Conditions in the experimental rearing room were maintained at 28 ± 2 °C, 60–70% RH and a photoperiod of L12:D12. The developmental time of larvae to fifth instar stage in each treatment was recorded. Thereafter, the larvae from each rearing tray were harvested and weighed to determine the yield per tray.

The amount of substrate consumed through the larval developmental phase and the frass produced were used to determine the waste degradation efficiency on dry weight basis (Eq 1).

$$\%Waste\;degradation=\frac{\left(Initial\;weight-Finial\;weight\right)}{Initial\;weight}\times 100$$ (1)

Biomass conversion rate (BCR), which indicates the efficiency of conversion of waste by BSF larvae into usable energy, was calculated using Eq 2 [10]. Dry weights were determined by oven drying at 105 °C for 24 hours.

$$\%BCR=[\frac{M_{PP}-M_{i}}{F_{in}}]\times 100$$ (2)

Where,

*M*_*i*_ is the dry weight of the larvae at start of experiment,

*M*_*pp*_ is the dry weight of larvae at 5^th^ instar,

*F*_*in*_ is the dry weight of the rearing substrates used.

The fraction of initial N accumulated in BSF larvae (BSFL) biomass was determined using Eq 3, while the fraction of initial N retained in the raw frass and mature frass fertilizer were determined using Eq 4.

$$\%N\;accumulated\;in\;BSFL\;biomass=\frac{(\frac{\%N\;in\;BSFL}{100})\times Dry\;BSFL\;yield}{Initial\;N\;content\;in\;substrate}\times 100$$ (3)

$$\%N\;retained=\frac{(\frac{\%N}{100})\times dry\;matter}{Initial\;N\;content\;in\;substrate}\times 100$$ (4)

Where,

$$Initial\;N\;content\;in\;substrate=\frac{\%N\;in\;formulated\;substrate\times dry\;weight\;of\;substrate}{100}$$

Dry matter represents the dry weight of raw frass or mature frass fertilizer.

### Frass fertilizer yield and quality

Frass from the experiments above was returned to their respective rearing trays and the process was managed using standard composting procedures until maturity. During the composting process, daily temperatures were recorded using a digital composting thermometer (Smart Choice; ASIN: B06XPCSCBW; USA). The metallic probe of the thermometer was inserted to a depth of 7 cm at four different sites of equal distance in each tray. The samples from each treatment were collected weekly to monitor compost maturity based on standard thresholds which included the ratio of carbon-to-nitrogen (C/N ratio, < 20) [44], electrical conductivity (< 4 mS cm^-1^) [45], pH (6–8), ammonium concentration (< 400 mg kg^-1^) [46], and germination index (> 80%) [47].

### Laboratory analysis methods

Laboratory-based analysis of compost pH and EC was carried out weekly using aqueous extracts of 1:10 (w/v) compost to distilled water. The contents were then shaken for 30 min at 180 revolutions min^-1^ on an orbital and linear shaker (MI0103002, Foure’s scientific, China). The pH and EC were then read directly using a pH (AD1000, Adwa, Romania) and EC meter (AVI, Labtech, India), respectively [41]. The nitrate and ammonium were extracted from compost using 0.5 M potassium sulphate at a ratio of 1:10 (w/v). Thereafter, the entire content of compost-potassium sulphate mixture was shaken for 1 hour using an orbital and linear shaker (KOS– 3333/KCS– 3333, MRC, UK) as described above. The solution was later filtered through a Whatman No. 1 filter paper and the filtrate was used for further analyses. Furthermore, the nitrate and ammonium concentrations were determined by colorimetric methods as described by Okalebo et al. [41]

At maturity, the compost from each treatment was weighed and oven-dried at 105 °C for 24 hours to remove moisture and establish the dry compost yield. In addition, 50 g of sub-samples of fresh compost from each treatment were collected, air-dried for five days and ground into powder that was used to determine the total organic carbon, N, P, K and C/N ratio bi-weekly for a period of five weeks (week 1, 3 and 5). Total organic carbon was determined using the wet oxidation method [48], while total N, P, K, (Ca and Mg were extracted using acid digestion [41]. From this extract, total N, P and K were determined using the Kjeldahl distillation method [49], UV-Vis spectrophotometry [41] and flame photometry [41], respectively, while Ca and Mg concentrations were determined using atomic absorption spectrometry [41] at 422.7 and 285.2 nm, respectively (iCE 3300 AA system, Thermo scientific, China).

### Phytotoxicity test on mature frass fertilizers

The germination index was determined by placing 10 cabbage seeds on filter papers moistened with 10 ml of 10% frass fertilizer extracts from each treatment in petri dishes for a duration of 96 hours at 25 °C under dark chamber conditions. The same procedure was repeated using distilled water as a positive control. Germinated seeds were counted, and their radicle lengths measured. Germination index (GI) was calculated using Eq 5 [50]. Frass fertilizers with GI values below 50% were considered highly phytotoxic, while values between 50% and 80% were moderately phytotoxic; and values above 80% indicated no phytotoxicity [47,50].

$$GI=\frac{\left(RSG\%\times RRG\%\right)}{100}$$ (5)

Where,

RSG (%) represents the relative seed germination calculated as:

$$RSG=\frac{number\;of\;seeds\;germinated\;in\;compost\;extracts}{number\;of\;seeds\;germinated\;in\;control(distilled\;water)}\times 100$$

RRG (%) represents the relative root growth calculated as:

$$RRG=\frac{mean\;root\;length\;in\;compost\;extract}{mean\;root\;length\;in\;control(distilled\;water)}\times 100$$

### Data analysis

Analysis of variance tests were performed on pH, electrical conductivity, ammonium, nitrate, ammonium/nitrate ratio, total organic carbon, C/N ratio, total N, P and K data using a linear mixed-effect model with ‘lmer’ function from the package ‘lme4’ in R statistical software with substrate and sampling time as fixed effects and replication as random effect. Data on fresh and dry BSF larval yields, waste reduction, biomass conversion rate, frass fertilizer yield, seed germination, germination index, initial substrate N content, N uptake by BSF larvae, N retention in frass were analysed using one-way analysis of variance test. Computation of least squares means was done using ‘lsmeans’ package, followed by mean separation using adjusted Tukey’s method implemented using ‘cld’ function from the ‘multicompView’ package. Data normality was assessed using Shapiro-Wilk test. Data on pH were transformed into hydrogen ion concentration before analysis. Principal component analysis was performed using the ‘prcomp’ function from the ‘ggbiplot’ package to determine the effect of biochar and gypsum substrate amendments on BSF larval yield, nitrogen utilisation and frass fertilizer quality. Data was analysed separately for each set of experiments. All the statistical analyses were conducted using R software version 3.6.0 [51].

## Results

### Black soldier fly larval yield

**Fig 1:**
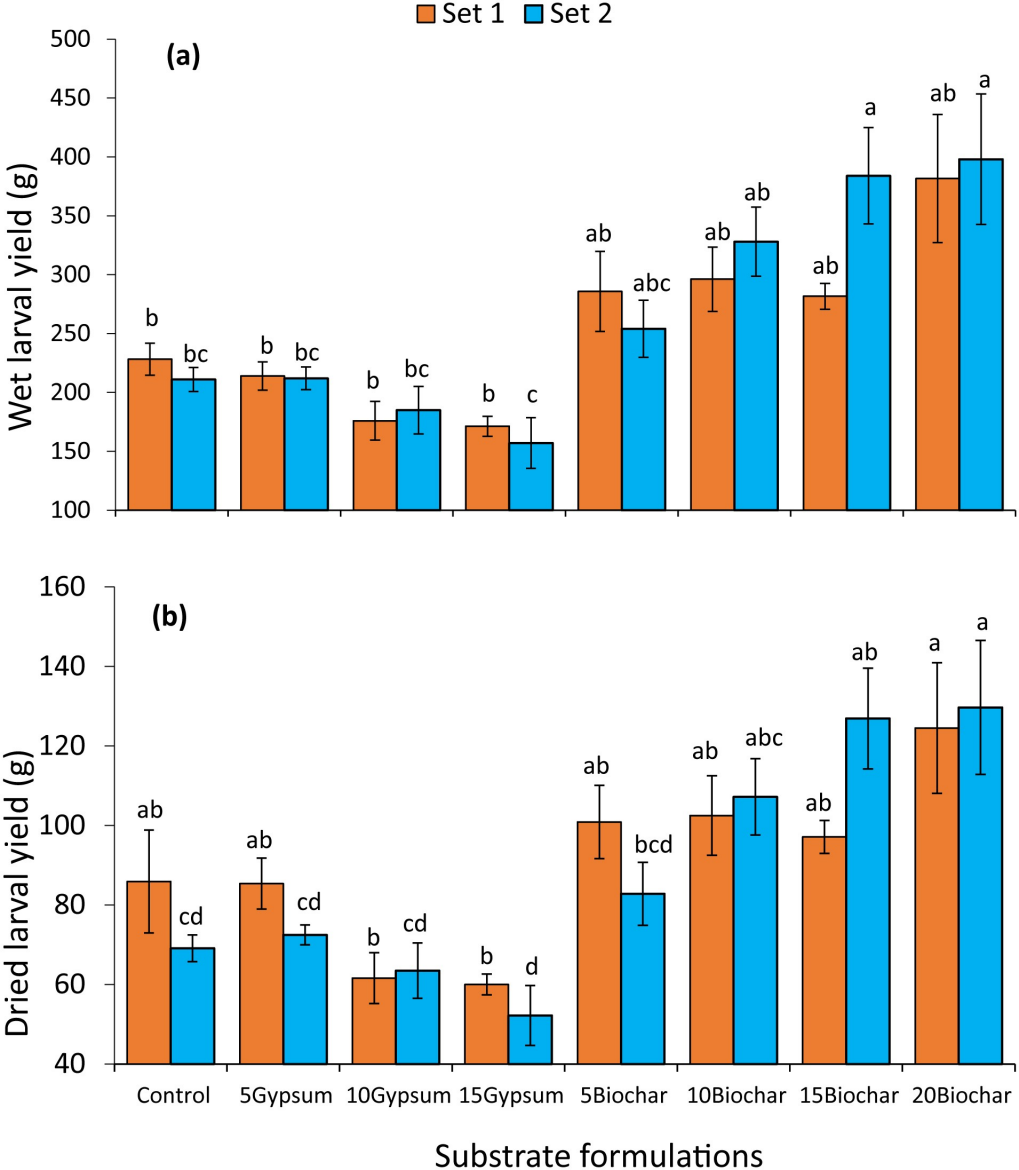
Effect of biochar and gypsum amendments on wet (a) and dry (b) black soldier fly larval yields during experiment set 1 and 2. **Key**: 5Gypsum, 10Gypsum, 15Gypsum = substrate amended with 5, 10 and 15% gypsum, respectively, 5Biochar, 10Biochar, 15Biochar and 20Biochar = substrate amended with 5, 10, 15 and 20% biochar, respectively, control = unamended substrate.

There was a significant effect of rearing substrates on the wet (experiment set 1: F = 7.088, df = 7, 16, p < 0.001, experiment set 2: F = 9.35, df = 7, 16, p < 0.001) and dry (experiment set 1: F = 5.107, df = 7, 16, p = 0.0033, experiment set 2: F = 9.697, df = 7, 16, p < 0.001) yields of BSF larvae (Fig 1a and 1b). Substrates amended with 20% biochar produced significantly higher wet (experiment set 1: p < 0.001, experiment set 2: p < 0.001) and dry (experiment set 1: p < 0.001, experiment set 2: p = 0.0033) larval yields than the control and gypsum treatments in both experiments. Amendment of rearing substrate using biochar significantly increased the wet and dry BSF larval yields by up to 89 and 85%, respectively, compared to the control substrate. Contrarily, substrates amended with gypsum caused decrease in dry larval yields of between 0.6 and 30%, although the decrease was not statistically significant. The highest decline in dry larval yield was recorded on substrates amended with 15% gypsum.

### Waste degradation, biomass conversion rates and frass fertilizer yield

Waste degradation efficiency in experiment set 1 (F = 6.639, df = 7, 16, p < 0.001) and set 2 (F = 18.17, df = 7, 16, p < 0.001) varied significantly (Table 2). The rate of waste degradation among the different treatments ranged between 22 and 57%. However, larvae fed on the control substrate had significantly higher (experiment set 1: p < 0.001, experiment set 2: p < 0.001) waste degradation efficiency than those provided with substrates amended with > 5% gypsum and > 10% biochar, except for 15% biochar in experiment set 1 (Table 2).

**Table 2:** Waste degradation, biomass conversion rates and frass fertilizer yield by black soldier fly larvae reared on biochar and gypsum amended substrates.

Substrate Formulations	Experiment set 1			Experiment set 2		
	Waste degradation efficiency (%)	Biomass conversion rate (%)	Frass fertilizer yield (kg)	Waste degradation efficiency (%)	Biomass conversion Rate (%)	Frass fertilizer yield (kg)
Control	57.4 ± 1.1a	7.4 ± 1.08b	0.49 ± 0.02e	40.6 ± 0.31a	8.2 ± 0.5cd	0.75 ± 0.02d
5Gypsum	53.0 ± 1.9ab	8.0 ± 0.82b	0.58 ± 0.01de	36.0 ± 0.76ab	9.7 ± 0.3cd	0.89 ± 0.04cd
10Gypsum	44.0 ± 1.2bc	6.9 ± 0.78b	0.79 ± 0.03bc	30.4 ± 0.91bc	9.9 ± 0.9cd	1.03 ± 0.03abc
15Gypsum	42.7 ± 0.84bc	6.9 ± 0.21b	0.85 ± 0.01b	22.9 ± 1.60d	10.8 ± 1.4cd	1.12 ±0.02ab
5Biochar	52.8 ± 2.9abc	9.6 ± 1.40ab	0.59 ± 0.04de	37.2 ± 0.91ab	10.8 ± 1.1cd	0.81 ± 0.04d
10Biochar	45.0 ± 4.0bc	11.5 ± 1.61ab	0.63 ± 0.02cde	36.8 ± 0.56ab	14.2 ± 1.1bc	0.89 ± 0.03cd
15Biochar	47.9 ± 0.29abc	10.0 ± 0.43ab	0.68 ± 0.02bcd	32.9 ± 1.04bc	18.2 ± 1.4b	0.97 ± 0.01bc
20Biochar	42.2 ± 2.9c	14.6 ± 1.68a	1.13 ± 0.08a	26.3 ± 3.10cd	24.2 ± 1.3a	1.23 ± 0.04a
p value	< 0.001	0.0021	< 0.001	< 0.001	< 0.001	< 0.001

**Key**: control = unamended substrate, 5Gypsum, 10Gypsum, 15Gypsum = substrate amended with 5, 10 and 15% gypsum, respectively; 5Biochar, 10Biochar, 15Biochar and 20Biochar = substrate amended with 5, 10, 15 and 20% biochar, respectively.

Significant differences were observed in BCR among the various treatments in experiment set 1 (F = 5.585, df = 7, 16, p = 0.0021) and set 2 (F = 25.85, df = 7, 16, p < 0.001) (Table 2). Larvae provided with substrates amended with 20% biochar had around double the BCR recorded in the control and significantly (experiment set 1: p = 0.0021, experiment set 2: p < 0.001) higher BCR than those reared on the control substrate and substrates amended with gypsum, but not for larvae reared on substrates amended with 10 and 15% biochar in experiment set 2. The lowest BCRs were recorded from larvae fed on substrates treated with 10 and 15% gypsum.

The frass fertilizer yields varied significantly in both experiment set 1 (F = 31.14, df = 7, 16, p < 0.001) and set 2 (F = 19.29, df = 7, 16, p < 0.001) (Table 2). Substrates amended with > 5% gypsum and > 10% biochar generated significantly (experiment set 1: p < 0.001, experiment set 2: p < 0.001) higher frass fertilizer yields than the control treatment.

### Nitrogen retention and uptake by black soldier fly larvae

At the start of the experiments, the initial total N content of substrate amended with biochar and gypsum were significantly different (F = 91.31, experiment set 1: df = 7, 16, p < 0.001, experiment set 2: F = 11.78, df = 7, 16, p < 0.001) (Table 3). The control treatment had significantly (experiment set 1: p < 0.001, experiment set 2: p < 0.001) higher initial total N content than substrates treated with various inclusion levels of biochar and gypsum. The quantity of total N accumulated in larval biomass varied significantly in experiment set 1 (F = 5.86, df = 7, 16, p = 0.0017) and experiment set 2 (F = 8.82, df = 7, 16, p < 0.001) (Table 3). Larvae fed on substrates amended with 20% biochar accumulated 2- and 3-times greater total N (experiment set 1: p = 0.0017, experiment set 2: p < 0.001) than those fed on control substrates in experiment set 1 and set 2, respectively.

**Table 3:** Effect of biochar and gypsum amendments on substrate nitrogen retention and uptake by black soldier fly larvae.

Substrate formulations	Experiment set 1				Experiment set 2			
	Initial N content in substrate (g)	Fraction of initial N accumulated in larval biomass (g/100g)	Fraction of initial N retained in raw frass (g/100g)	N content in frass fertilizer as % of initial N content (g/ 100g)	Initial N content in substrates (g)	Fraction of initial N accumulated in larval biomass (g/ 100g)	Fraction of initial N retained in raw frass (g/ 100g)	N content in frass fertilizer as % of initial N content (g /100g)
Control	52.0 ± 0.80a	4.8 ± 0.59b	37.1 ± 2.93	23.4 ±1.4d	43.1 ± 1.32a	4.8 ± 0.40c	55.0 ± 4.02c	33.6 ± 0.9c
5Gypsum	44.0 ± 0.75bc	6.9 ± 0.81b	49.0 ± 5.48	28.7 ± 1.6d	36.2 ± 0.64bc	6.1 ± 0.35bc	68.6 ± 2.89abc	47.5 ± 2.1ab
10Gypsum	43.1 ± 0.00c	4.8 ± 0.31b	50.7 ± 1.37	39.9 ± 3.4bc	35.2 ± 0.06bc	5.5 ± 0.64bc	73.9 ± 2.88ab	53.7 ± 2.8a
15Gypsum	39.8 ± 0.56d	5.1 ± 0.43b	56.1 ± 1.40	41.5 ± 1.7b	33.5 ± 0.72c	4.8 ± 0.58c	83.3 ± 2.88a	52.2 ± 3.0ab
5Biochar	46.4 ± 0.28b	7.2 ± 1.53ab	39.1 ± 0.95	25.2 ± 0.6d	38.4 ± 0.50ab	6.9 ± 1.05bc	54.2 ± 0.85c	40.7 ± 3.5abc
10Biochar	43.6 ± 0.53c	8.3 ± 0.95ab	51.5 ± 8.45	28.1 ± 0.4d	38.8 ± 0.18ab	6.1 ± 0.01bc	51.2 ± 0.84c	39.5 ±3.0bc
15Biochar	44.9 ± 0.64bc	6.4 ± 0.54b	43.3 ± 3.35	30.3 ± 1.4cd	32.6 ± 2.12c	9.4 ± 1.21ab	64.4 ± 6.97abc	48.1 ± 2.8ab
20Biochar	34.1 ± 0.16e	11.6 ± 1.45a	44.7 ± 2.28	56.2 ± 3.7a	36.3 ± 0.19bc	11.4 ± 1.13a	62.2 ± 5.37bc	49.4 ± 3.5ab
p value	< 0.001	0.0017	0.0529	< 0.001	< 0.001	< 0.001	< 0.001	0.0013

**Key**: control = unamended substrate, 5Gypsum, 10Gypsum, 15Gypsum = substrate amended with 5, 10 and 15% gypsum, respectively, 5Biochar, 10Biochar, 15Biochar and 20Biochar = substrate amended with 5, 10, 15 and 20% biochar, respectively.

The fraction of initial total N retained in the raw frass varied significantly among the various substrates in experiment set 2 (F = 8.076, df = 7, 16, p < 0.001) but not in experiment set 1 (F = 2.613, df = 7, 16, p = 0.0529). In experiment set 2, amendment of substrates with 15% gypsum conserved significantly (p < 0.001) higher total N in frass than in biochar and control treatments, except for 15% biochar (Table 3). The fraction of initial total N retained in mature frass fertilizer also varied significantly (experiment set 1: F = 27.32, df = 7, 16, p < 0.001, experiment set 2: F = 6.15, df = 7, 16, p = 0.0013). Frass fertilizer from substrates amended with 20% biochar retained significantly (p < 0.001) higher total N content than other treatments in experiment set 1. In experiment set 2, the frass of initial total N retained in frass fertilizers from substrates amended with gypsum, and those amended with 15 and 20% biochar was significantly (p = 0.0013) higher than that retained in frass fertilizer produced from the unamended substrate.

### Effect of biochar and gypsum on temperature, pH, and electrical conductivity during frass composting

**Fig 2:**
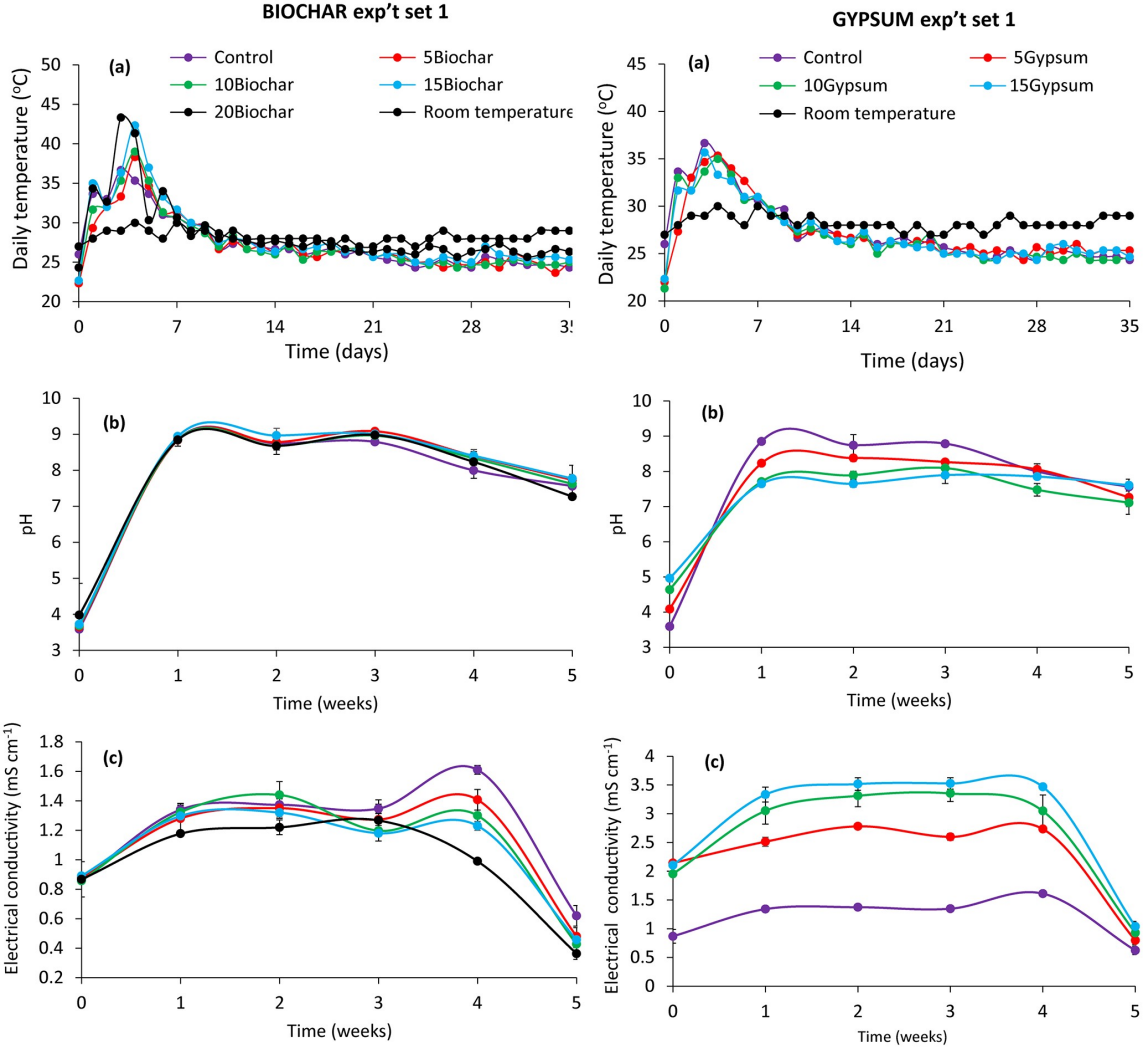
Changes in temperature (a), pH (b) and electrical conductivity (c) during experiment set 1 of BSF frass composting. **Key**: 5Gypsum, 10Gypsum, 15Gypsum = substrate amended with 5, 10 and 15% gypsum, respectively, 5Biochar, 10Biochar, 15Biochar and 20Biochar = substrate amended with 5, 10, 15 and 20% biochar, respectively, control = unamended substrate.

**Fig 3:**
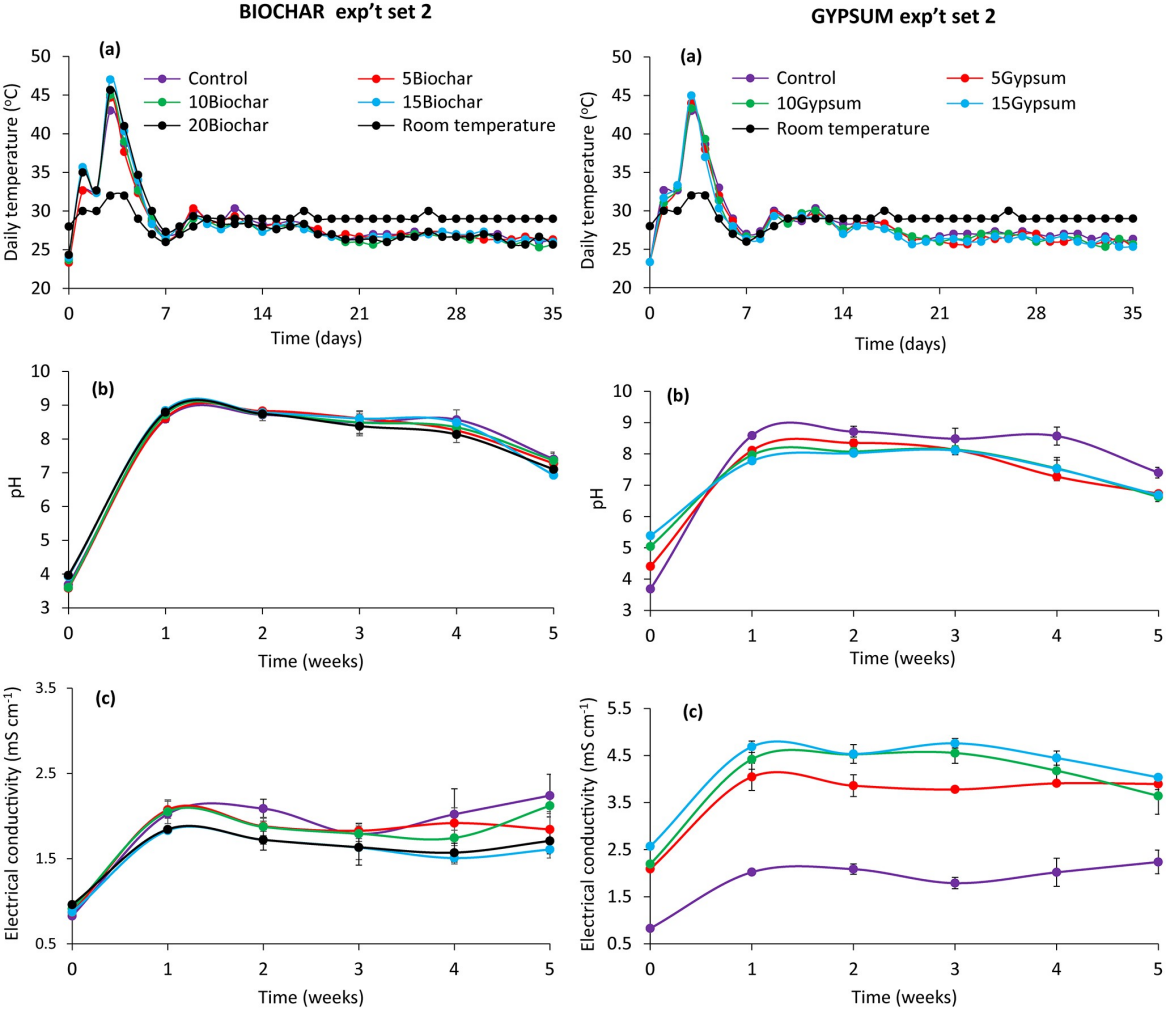
Changes in temperature (a), pH (b) and electrical conductivity (c) during experiment set 2 of BSF frass composting. **Key**: 5Gypsum, 10Gypsum, 15Gypsum = substrate amended with 5, 10 and 15% gypsum, respectively, 5Biochar, 10Biochar, 15Biochar and 20Biochar = substrate amended with 5, 10, 15 and 20% biochar, respectively, control = unamended substrate.

The variation in temperature during the BSF rearing and composting phases is presented in Figs 2a and 3a. The temperature during this period in both experiments peaked between 43–47 °C on the third and fourth day of BSF rearing. The highest temperatures were recorded for substrates amended with 20% biochar (43 °C) and 15% biochar (47 °C) on the third day (Figs 2a and 3a) for experiments set 1 and 2, respectively. A sharp decline of temperature was then observed up to the ninth day (28–30 °C). Thereafter, temperature fluctuations were negligible until the end of the experiments.

The pH was significantly affected by substrate amendments (experiment set 1: χ^2^ = 112, df = 7, p < 0.001, experiment set 2: χ^2^ = 54.19, df = 7, p < 0.001) and composting time (experiment set 1: χ^2^ = 5817.97, df = 5, p < 0.001, experiment set 2: χ^2^ = 4625.80, df = 5, p < 0.001). The interaction of substrate amendments and composting time was also significant (experiment set 1: χ^2^ = 267.51, df = 35, p < 0.001, experiment set 2: χ^2^ = 275.24, df = 35, p < 0.001) during experiments (Figs 2b and 3b). The pH significantly increased in the first week and reached peak values between the first and third week of composting, ranging between 7.9 and 9.1. Substrate treated with gypsum had the lowest pH values throughout the rearing and composting process in both experiments. On the other hand, substrates amended with 15% biochar inclusion had the highest pH value (7.8) at the end of composting process.

The EC of the various treatments also varied significantly due to substrate amendments (experiment set 1: χ^2^ = 3208.18, df = 7, p, 0.001 experiment set 2: χ^2^ = 2608, df = 7, p < 0.001) and composting time (experiment set 1: χ^2^ = 1554.61, df = 5, p < 0.001, experiment set 2: χ^2^ = 549.72, df = 5, p < 0.001). The interaction was also significant (χ^2^ = 450.45, df = 35, p < 0.001, experiment set 2: χ^2^ = 121.69, df = 35, p < 0.001). The pattern of EC fluctuation throughout the experiments is shown in Figs 2c and 3c. Gypsum amended substrates had significantly (p < 0.001) higher EC values compared to biochar amended substrates throughout the study. However, in both experiments (set 1 and set 2), the EC values of substrates not amended with gypsum were significantly (p < 0.001) much lower. In mature frass composts, substrates amended with 15% gypsum had the highest EC value (4 mS cm ^-1^), while those amended with 20% biochar had the lowest EC value (0.36 mS cm^-1^).

#### Ammonium and nitrate concentrations during frass composting

**Fig 4:**
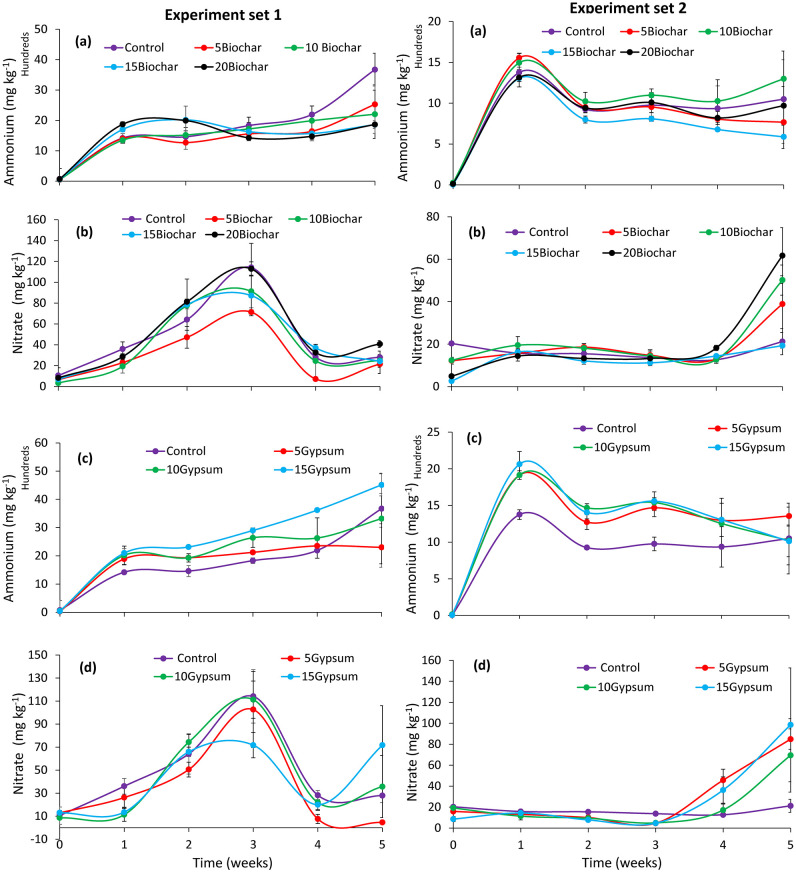
Trends of ammonium (a and c) and nitrate (b and d) concentrations during BSF frass composting. **Key**: 5Gypsum, 10Gypsum, 15Gypsum = substrate amended with 5, 10 and 15% gypsum, respectively, 5Biochar, 10Biochar, 15Biochar and 20Biochar = substrate amended with 5, 10, 15 and 20% biochar, respectively, control = unamended substrate.

Ammonium concentration was significantly influenced by the substrate treatments (experiment set 1 χ^2^ = 51.96, df = 7, p < 0.001, experiment set 2: χ^2^ = 70.88, df = 7, p < 0.001), composting time (experiment set 1: χ^2^ = 247.32, df = 5, p < 0.001, experiment set 2: χ^2^ = 450.584, df = 5, p < 0.001), but not their interactions (experiment set 1: χ^2^ = 46.717, df = 35, p = 0.089, experiment set 2: χ^2^ = 31.97, df = 35, p = 0.615) (Fig 4a and 4c). The ammonium concentration in all the treatments (*i*.*e*. ‘control’, biochar and gypsum amended substrates) had a similar pattern of increase in both experiments. Ammonium concentration significantly (p < 0.001) increased in the first week. Gypsum amended substrates retained higher ammonium concentration than biochar treatments. In mature composts, the ammonium concentration ranged between 588 and 4516 mg kg^-1^ in all the treatments. However, the highest ammonium concentration was achieved when substrates were amended with 15% gypsum.

The nitrate concentration varied significantly due substrate amendments with biochar and gypsum (χ^2^ = 14.34, df = 7, p = 0.0455) in experiment set 1 only and composting time (experiment set 1: χ^2^ = 319.87, df = 5, p < 0.001, experiment set 2: χ^2^ = 110.95, df = 5, p < 0.001) in both experiments. The interaction of substrate and composting time was significant during experiment set 2 only (χ^2^ = 57.483, df = 35, p < 0.001) (Fig 4b and 4d). The peak concentrations of nitrate were observed in the 3^rd^ and 5^th^ weeks of experiment set 1 (p < 0.001) and set 2 (p < 0.001), respectively. For both experiments (set 1 and set 2), the highest concentration of nitrates in mature frass fertilizers was recorded in substrates amended with 15% gypsum.

### Total organic carbon, nitrogen, phosphorus, and potassium during frass composting

**Fig 5:**
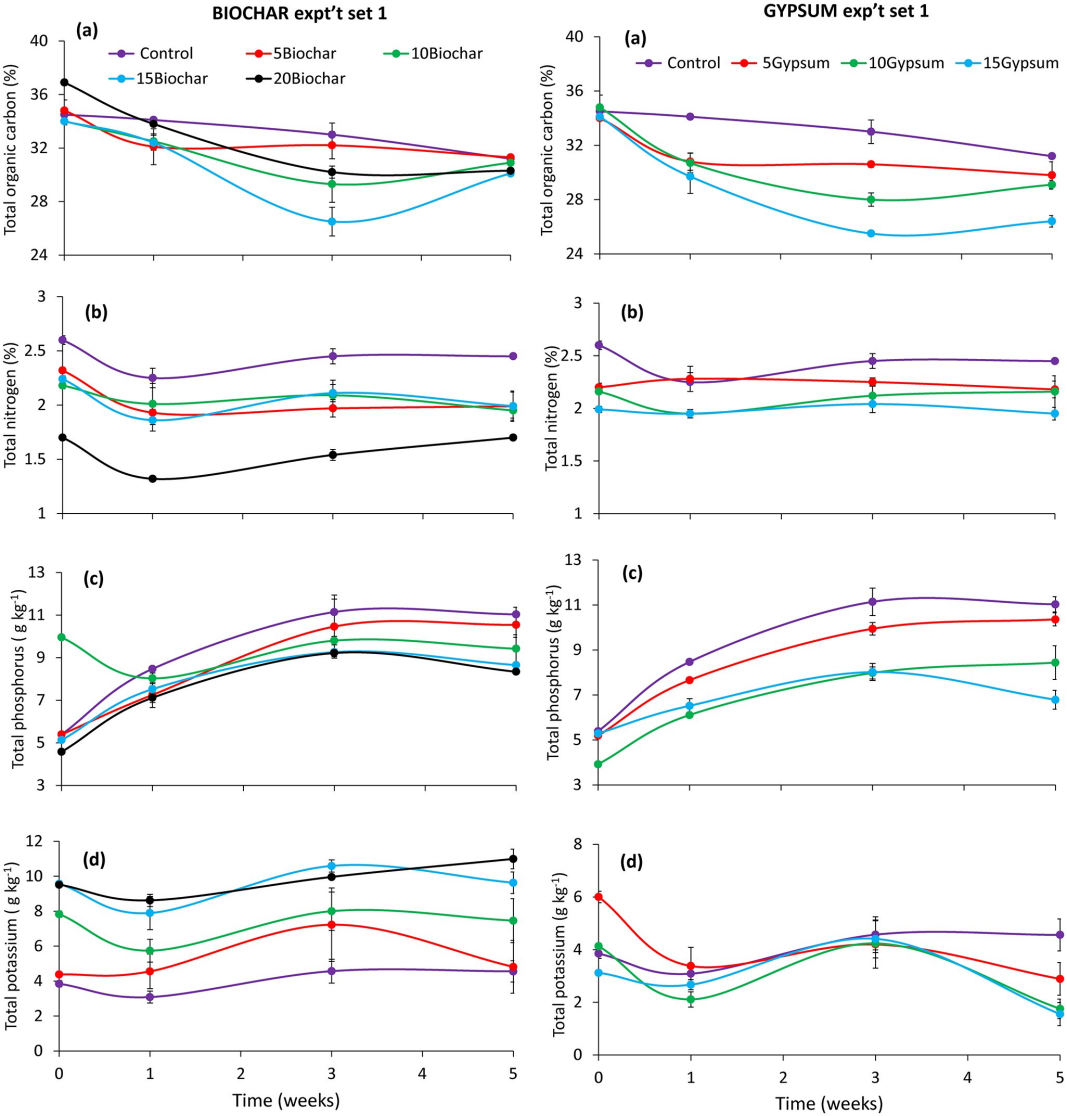
Changes in concentrations of total organic carbon (a), nitrogen (b), phosphorus (c) and potassium (d) during experiment set 1 of BSF frass composting. **Key**: 5Gypsum, 10Gypsum, 15Gypsum = substrate amended with 5, 10 and 15% gypsum, respectively, 5Biochar, 10Biochar, 15Biochar and 20Biochar = substrate amended with 5, 10, 15 and 20% biochar, respectively, control = unamended substrate.

**Fig 6:**
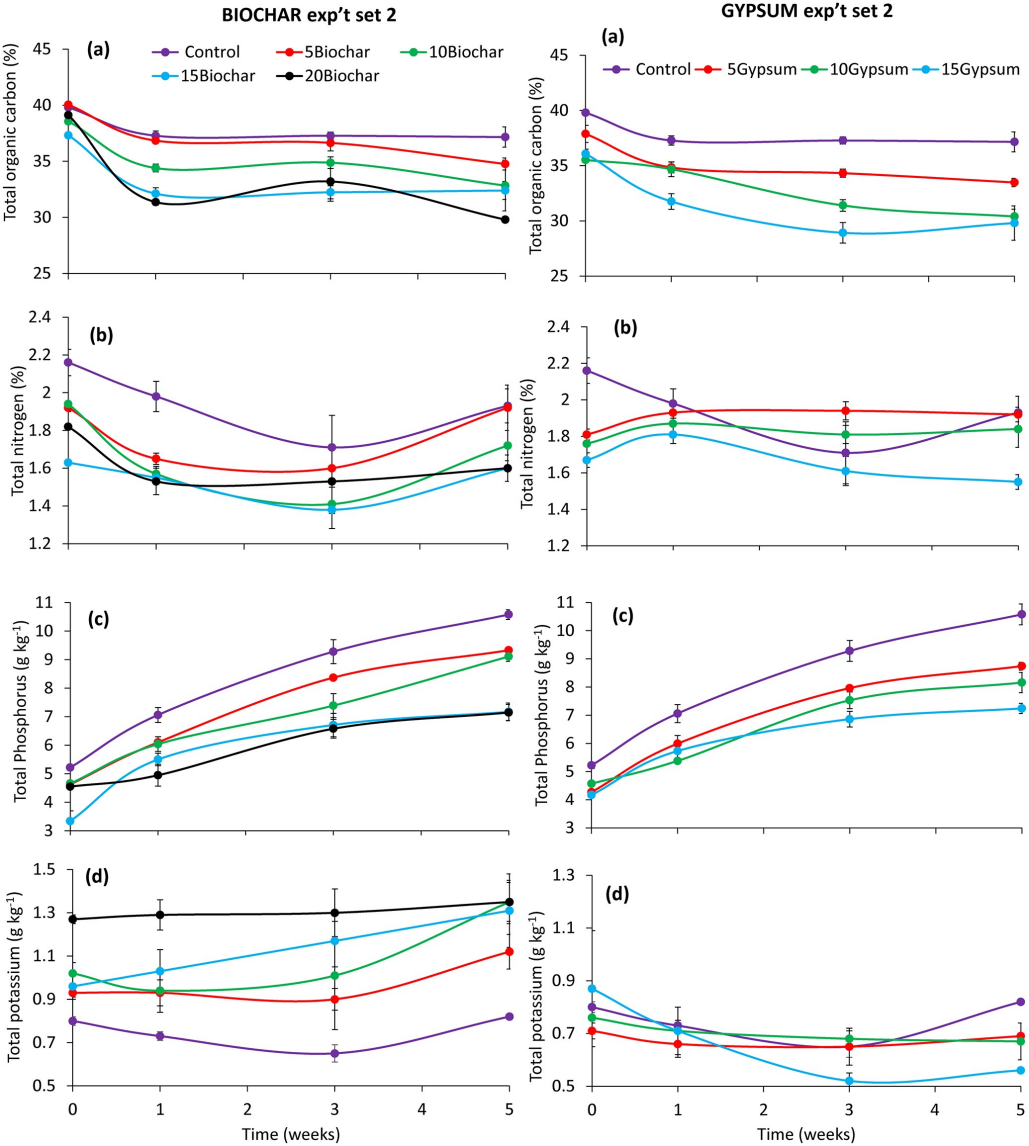
Changes in concentrations of total organic carbon (a), nitrogen (b), phosphorus (c) and potassium (d) during experiment set 2 of BSF frass composting. **Key**: 5Gypsum, 10Gypsum, 15Gypsum = substrate amended with 5, 10 and 15% gypsum, respectively, 5Biochar, 10Biochar, 15Biochar and 20Biochar = substrate amended with 5, 10, 15 and 20% biochar, respectively, control = unamended substrate.

There was a significant impact of substrates (experiment set 1: χ^2^ = 131.87, df = 7, p < 0.001, experiment set 2: χ^2^ = 210.79, df = 7, p < 0.001), composting time (experiment set 1: χ^2^ = 328.617, df = 3, p < 0.001, experiment set 2: χ^2^ = 228.94, df = 3, p < 0.001), and their interaction (experiment set 1: χ^2^ = 81.94, df = 21, p < 0.001, experiment set 2: χ^2^ = 49.26, df = 21, p < 0.001) on total organic carbon concentration during frass composting. There was a gradual decrease in total organic carbon concentration in compost treatments throughout both experiments (Figs 5a and 6a). Significant decreases (p < 0.001) in total organic carbon were observed in the first and third weeks of experiments. Compost treatments with biochar inclusion had higher total organic carbon concentrations than gypsum amended treatments.

The total N concentration also varied significantly due to substrate treatments (experiment set 1: χ^2^ = 257.45, df = 7, p < 0.001, experiment set 2: χ^2^ = 92.88, df = 7, p < 0.001) composting time (experiment set 1: χ^2^ = 32.725, df = 3, p < 0.001, experiment set 2: χ^2^ = 30.05, df = 3, p < 0.001) during experiments. The interaction effect was only significant in experiment set 2 (χ^2^ = 48.54, df = 21, p < 0.001). The N concentration reduced in the first and third week during experiment set 1 and 2, respectively. The control treatments generated frass fertilizer with the highest total N concentrations (Figs 5b and 6b). However, compost obtained from substrates amended with 5% gypsum or 5% biochar had higher values of total N than the other treatments.

Total P concentration during experiments, varied significantly due to substrate treatment amendment (experiment set 1: χ^2^ = 180, df = 7, p < 0.001, experiment set 2: χ^2^ = 231.08, df = 7, p < 0.001), composting time (experiment set 2: χ^2^ = 485.91, df = 3, p < 0.001, experiment set 2: χ^2^ = 1048.08, df = 3, p < 0.001) and their interaction (experiment set 1: χ^2^ = 131.46, df = 21, p < 0.001, experiment set 2: χ^2^ = 61.23, p < 0.001). There were significant (p < 0.001) increases in total P in the first and third weeks of experiment set 1, and throughout experiment set 2 (Figs 5c and 6c). Amendment of substrates with 5% of either biochar or gypsum produced frass fertilizers with higher total P concentration than the other amended treatments. The frass fertilizer generated from the control treatments had higher P concentrations than those of biochar and gypsum amended substrates.

Total K concentrations of the different compost treatments were also significantly affected by substrate amendments (experiment set 1: χ^2^ = 539.43, df = 7, p < 0.001, experiment set 2: χ^2^ = 211.27, df = 7, p < 0.001) and frass composting time (experiment set 1: χ^2^ = 40.03, df = 3, p < 0.001, experiment set 2: χ^2^ = 9.54, df = 3, p = 0.0229) during experiments. The interaction effect was significant in experiment set 1 only (χ^2^ = 40.54, df = 21, p = 0.0064) (Figs 5d and 6d). Biochar amended composts had higher K concentrations than the control as well as gypsum treated composts. The concentration of potassium in the compost was observed to increase with increased biochar inclusion rates, while potassium concentrations decreased with increased gypsum inclusion ratios.

### Ratios of carbon to nitrogen during frass composting

The ratios of total organic carbon to total nitrogen were significantly affected by substrate amendments (experiment set 1: χ^2^ = 336.51, df = 7, p < 0.001, experiment set 2: χ^2^ = 33.59, df = 7, p < 0.001) and composting time (experiment set 1: χ^2^ = 72.0, df = 3, p < 0.001, experiment set 2: χ^2^ = 18.72, df = 3, p < 0.001). The interaction of substrate treatments and composting time was significant in experiment set 1 only (χ^2^ = 86.09, df = 21, p < 0.001) (Table 4). At the start of the experiments, substrates treated with 15% biochar had the highest C/N ratio, while the ‘control’ substrate was found to have the lowest C/N ratio. Minimal reduction in C/N ratios were noted across all treatments tested. In mature frass fertilizers, biochar amended substrates had higher C/N ratios than gypsum amended and ‘control’ treatments. At the end of the composting process, substrates amended with 15% biochar had the highest C/N ratio (20.3) (experiment set 2), while the control treatment had the lowest value (12.8) (experiment set 1).

**Table 4:** Ratios of total organic carbon to total nitrogen contents at selected periods during frass composting.

Substrate formulations	Experiment set 1				Experiment set 2			
	Time (weeks)							
	0	1	3	5	0	1	3	5
Control	13.3 ± 0.33d	15.2 ± 0.48bc	13.5 ± 0.67b	12.8 ± 0.12b	18.5 ± 0.69b	18.8 ± 0.66bc	22.2 ± 2.28	19.3 ± 1.28
5Gpysum	15.4 ± 0.27c	13.6 ± 0.49c	13.6 ± 0.30b	13.7 ± 0.10b	20.9 ± 0.07ab	18.0 ± 0.49bc	17.7 ± 0.66	17.4 ± 0.48
10Gypsum	16.2 ± 0.43bc	15.7 ± 0.09bc	13.3 ± 0.79b	13.6 ± 1.09b	20.2 ± 0.03ab	18.6 ± 0.62bc	17.4 ± 0.75	16.6 ± 0.54
15Gypsum	17.1 ± 0.23bc	15.3 ± 0.65bc	12.5 ± 0.52b	13.6 ± 0.52b	21.6 ± 0.72ab	17.6 ± 0.55c	18.0 ± 1.23	19.3 ± 1.02
5Biochar	15.0 ± 0.43c	16.7 ± 1.23bc	16.4 ± 1.07ab	15.8 ± 1.06ab	20.9 ± 0.15ab	22.3 ± 0.46a	23.1 ± 0.95	18.2 ± 0.93
10Biochar	15.6 ± 0.19bc	16.4 ± 1.32bc	14.2 ± 1.41b	15.9 ± 0.62ab	19.9 ± 0.26ab	21.9 ± 0.21a	25.4 ± 3.85	19.3 ± 1.24
15Biochar	15.1 ± 0.33c	17.5 ± 0.95b	12.6 ± 0.72b	15.2 ± 1.02ab	23.1 ± 1.54a	20.9 ± 0.55ab	23.4 ± 1.17	20.3 ± 1.08
20Biochar	21.7 ± 0.07a	25.6 ± 0.12a	19.7 ± 0.33a	17.9 ± 0.15a	21.5 ± 0.23ab	20.6 ± 0.80abc	22.6 ± 4.00	18.7 ± 0.83
p value	< 0.001	< 0.001	< 0.001	0.0019	0.0079	< 0.001	0.148	0.235

**Key**: control = unamended substrate, 5Gypsum, 10Gypsum, 15Gypsum = substrate amended with 5, 10 and 15% gypsum, respectively, 5Biochar, 10Biochar, 15Biochar and 20Biochar = substrate amended with 5, 10, 15 and 20% biochar, respectively.

### Mature frass fertilizer quality

In the mature frass fertilizer treatments, the concentrations of total N (F = 5.109, experiment set 1: df = 7, 16, p = 0.0033, experiment set 2: F = 4.077, df = 7, 16, p = 0.0095), total P (experiment set 1: F = 9.282, df = 7, 16, p < 0.001, experiment set 2: F = 20.96, df = 7, 16, p < 0.001) and total K (experiment set 1: F = 17.95, df = 7, 16, p < 0.001 experiment set 2: F = 16.12, df = 7, 16, p < 0.001) varied significantly in both experiments (Table 5). The control treatment had significantly higher total N concentration than frass fertilizer generated from substrates amended with 15% gypsum, 10 and 20% biochar in experiment set 1. The same treatment significantly higher P concentrations in frass fertilizer than other treatments, except for frass fertilizer amended with 10 and 5% gypsum, and 10 and 20% biochar in experiment set 1, and 5% biochar in experiment set 2. Frass fertilizer amended with 15 and 20% biochar inclusion levels had significantly (p < 0.001) higher K concentrations than the other treatments, but not for amendment with 10% biochar in experiment set 1. In experiment set 2, frass fertilizer from substrates amended with 10–20% biochar achieved significantly (p < 0.001) higher K levels than those generated from the control and gypsum amended substrates.

**Table 5:** Concentrations of nitrogen (N), phosphorus (P) and potassium (K) in amended mature black soldier fly frass fertilizers.

Substrate formulations	Experiment set 1			Experiment set 2		
	N (%)	P (g kg^-1^)	K (g kg^-1^)	N (%)	P (g kg^-1^)	K (g kg^-1^)
Control	2.45 ± 0.02a	11.03 ± 0.34a	4.56 ± 0.62bc	1.94 ± 0.087a	10.58 ± 0.37a	0.82 ± 0.004bc
5Gypsum	2.18 ± 0.08ab	10.36 ± 0.29ab	2.89 ± 0.62c	1.92 ± 0.044a	8.74 ± 0.14b	0.69 ± 0.00c
10Gypsum	2.16 ± 0.15abc	8.44 ± 0.75bc	1.75 ± 0.37c	1.84 ± 0.102a	8.16 ± 0.36bc	0.67 ± 0.069c
15Gypsum	1.95 ± 0.05bc	6.79 ± 0.42c	1.55 ± 0.44c	1.55 ± 0.042a	7.24 ± 0.18c	0.56 ± 0.013c
5Biochar	1.99 ± 0.14abc	10.54 ± 0.58ab	4.82 ± 1.51bc	1.92 ± 0.120a	9.33 ± 0.25ab	1.12 ± 0.075ab
10Biochar	1.95 ± 0.07bc	9.42 ± 0.66ab	7.46 ± 1.26ab	1.72 ± 0.084a	9.11 ± 0.17b	1.35 ± 0.097a
15Biochar	1.99 ± 0.13abc	8.65 ± 0.10bc	9.63 ± 0.62a	1.60 ± 0.074a	7.17 ± 0.31c	1.31 ± 0.168a
20Biochar	1.70 ± 0.02c	8.34 ± 0.07bc	10.99 ± 0.56a	1.60 ± 0.069a	7.15 ± 0.29c	1.35 ± 0.085a
p value	0.0033	< 0.001	< 0.001	0.0095	< 0.001	< 0.001

**Key**: control = unamended substrate, 5Gypsum, 10Gypsum, 15Gypsum = substrate amended with 5, 10 and 15% gypsum, respectively, 5Biochar, 10Biochar, 15Biochar and 20Biochar = substrate amended with 5, 10, 15 and 20% biochar, respectively

### Seed germination and germination index of mature frass fertilizer extracts

The germination rate (experiment set 1: F = 0.731, df = 7, 16, p = 0.649, experiment set 2: F = 1.446, df = 7, 16, p = 0.255) and germination index (experiment set 1: F = 0.384, df = 7, 16, p = 0.899, experiment set 2: F = 2.332, df = 7, 16, p = 0.0762) of the cabbage seeds were not significantly influenced by the compost used for both experiments (Table 6). The seed germination rate was greater than 83% for all compost treatments. Frass fertilizers generated from biochar amended substrates had higher seed germination indices than the composts derived from the unamended substrate. Similarly, frass fertilizers from substrates amended with biochar had higher seed germination rates and germination indices (GI) than those amended with gypsum in experiment set 2.

**Table 6:** Seed germination and germination indices of mature frass fertilizers generated from substrates amended with biochar and gypsum.

Substrate formulations	Experiment set 1		Experiment set 2	
	Germination rate (%)	Germination index (%)	Germination rate (%)	Germination index (%)
Control	93.3 ± 3.3	90.2 ± 26.9	90 ± 5.8	232.2 ± 92.6
5Gypsum	90 ± 5.8	115.1 ± 13.8	93.3 ± 6.7	56.2 ± 7.4
10Gypsum	83.3 ± 6.7	120.8 ± 37.0	86.7 ± 6.7	101.1 ± 45.7
15Gypsum	86.7 ± 3.3	123 ± 19.5	63.3 ± 23.3	68.4 ± 29.2
5Biochar	83.3 ± 12.0	98.3 ± 29.9	100 ± 0.0	273.5 ± 51.8
10Biochar	93.3 ± 3.3	133.7 ± 15.5	90 ± 5.8	158.1 ± 29.8
15Biochar	93.3 ± 3.3	108.1 ± 11.6	100 ± 0.0	165.4 ± 61.4
20Biochar	96.7 ± 3.3	114.1 ± 9.5	90 ± 5.8	132 ± 30.2
p value	0.629	0.899	0.255	0.0762

**Key**: control = unamended substrate, 5Gypsum, 10Gypsum, 15Gypsum = substrate amended with 5, 10 and 15% gypsum, respectively, 5Biochar, 10Biochar, 15Biochar and 20Biochar = substrate amended with 5, 10, 15 and 20% biochar, respectively.

### Multivariate analysis of black soldier fly larval growth and frass fertilizer quality

**Fig 7:**
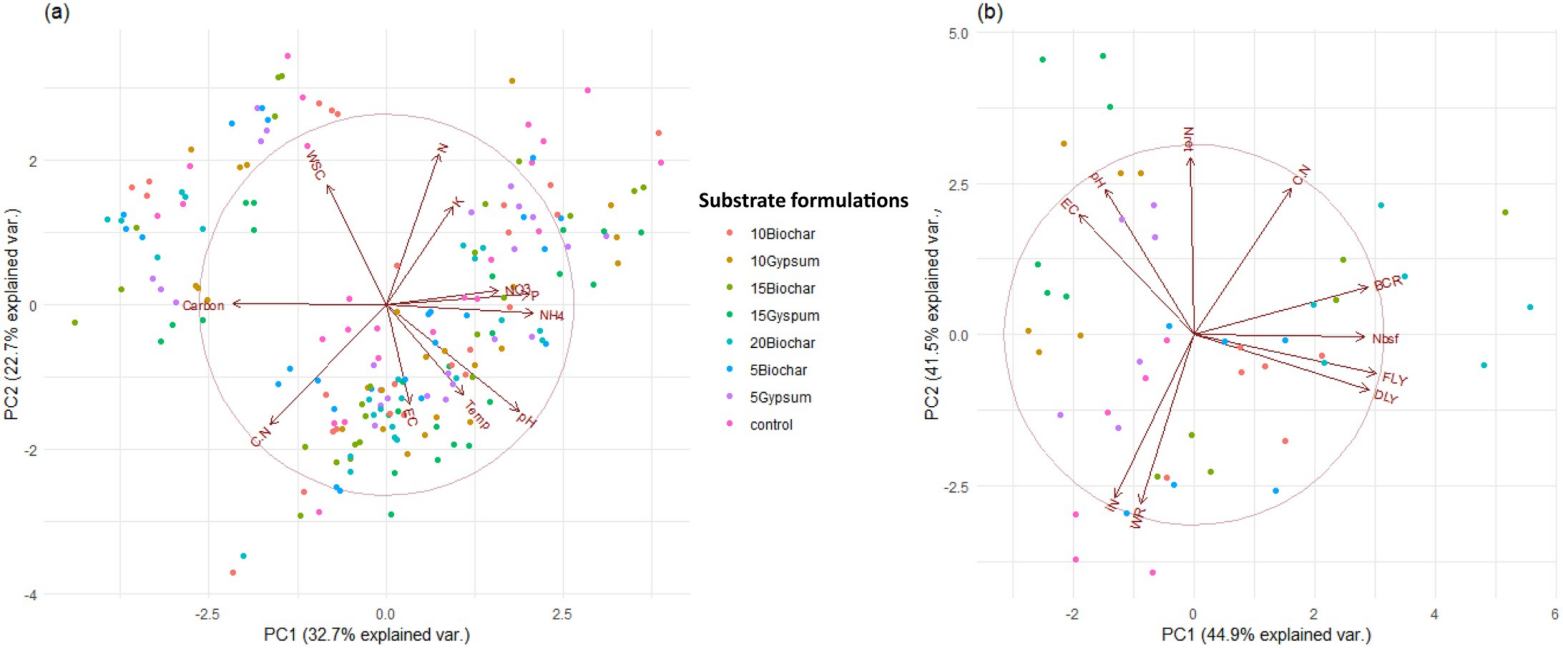
Bi-plot graphs based on PC analysis of parameters that measure frass fertilizer quality (a) and black soldier fly larval yields (b) as a function of the different biochar and gypsum amendments for nitrogen conservation in the rearing substrates. **Key**: NO_3_ = nitrate nitrogen, NH_4_ ammonium nitrogen, N = total nitrogen, K = total potassium, P = total phosphorus, C:N = carbon to nitrogen ratio, EC = electrical conductivity, Temp = temperature, WSC = water soluble carbon, iN = initial nitrogen content in the rearing substrates, Nbsf = nitrogen accumulated in BSF larval biomass, Nret = nitrogen retained in the frass, WR = waste degradation, BCR = biomass conversion rate, FLY = wet BSF larval yield, DLY = dry BSF larval yield. 5Gypsum, 10Gypsum, 15Gypsum = substrate amended with 5, 10 and 15% gypsum, respectively, 5Biochar, 10Biochar, 15Biochar and 20Biochar = substrate amended with 5, 10, 15 and 20% biochar, respectively, control = unamended substrate.

Principal component analysis (PCA) revealed that compost quality was significantly affected by biochar and gypsum amendment. The first two components of PCA explained 55.4% of the total data variance with PC 1 and PC 2 accounting for 32.7 and 22.7%, respectively (Fig 7a). There was a positive correlation between total N and K contents as well as between ammonium, nitrate, and P concentrations of the frass fertilizers. However, the substrate C/N ratio was observed to be negatively correlated with total N and K concentration of the frass fertilizers. Compost temperature, pH and electrical conductivity varied negatively with water soluble carbon of the frass fertilizers.

The total N intake, N retention in the frass and BSF larva yield (wet and dried weight larvae) were significantly affected by substrate amendment. Here, the PCA analysis explained 86.4% of the data variance, whereby PC 1 and PC 2 represented 44.9% and 41.5%, respectively, (Fig 7b). It was observed that the wet and dried BSF larval yields, substrate C/N ratio, biomass conversion rates and total N accumulated in larval biomass were positively correlated. However, the dried and wet BSF larval yields were negatively correlated with pH and EC of the rearing substrates. Waste degradation efficiency and the initial substrate N content were also negatively correlated with the C/N ratios of the rearing substrates.

## Discussion

### Effect of biochar and gypsum amendments on black soldier fly larval yields

The present study revealed higher yields of wet and dried BSF larvae from biochar-amended substrates, especially at inclusion levels of 15 and 20%. These findings are in line with those reported by Awash et al. [32,52] and Wang et al. [30], who demonstrated that improved nitrogen conservation can be achieved through biochar amendment of substrates. Similarly, Lalander et al. [1] established that BSF larvae fed on substrates with high nitrogen concentration developed faster and had higher biomass accumulation. Furthermore, Sanchez-Monedero et al. [29] also showed that biochar amendment has the ability to improve water holding capacity and porosity of substrate, which enhances larvae mobility and feeding. Several authors using nitrogen-rich substrates such as dog food, poultry food, abattoir waste, food remains, chicken, pig and cow manures have also reported higher BSF larval yields [1,8,9,53].

In contrast to biochar, gypsum amended substrates yielded less BSF larval biomass, despite its efficiency in nitrogen conservation in organic substrates [54]. This might be attributed to aggregation and drying properties of substrates induced by gypsum inclusion, which might have negatively influenced BSF growth performance. The BSF larvae have been reported to thrive in substrates with 70% moisture content [55]. Hence, the drying properties of substrates due to gypsum inclusion further explain the low larvae performance.

High EC of substrates amended with gypsum was observed in the current studies. These high EC levels are known to be directly related to high salt content, which has been documented to reduce nutrient uptake and assimilation by insect larvae [56]. However, further studies to evaluate the effects of high EC on BSF larval performance are warranted.

### Effect of biochar and gypsum amendments on frass fertilizer yield and quality

Throughout the experiments, substrates amended with biochar had lower waste degradation efficiency and hence higher frass fertilizer yields. This is consistent with previous findings reported by Manurung et al. [6] and Supriyatna et al. [7] when high recalcitrant carbon substrates with high C/N ratios such as rice straw and cassava peels were used for rearing BSF larvae. This is further supported by Khan et al [57] who demonstrated that substrates amended with carbon-rich materials such as sawdust and biochar were extremely difficult to be broken down. The factors that influenced the low frass fertilizer yields in unamended substrates (control treatment) compared to those amended with gypsum and biochar are likely due to the high waste degradation efficiency observed in control treatment. This makes biochar a better amendment for organic fertilizer production, since it also enhances frass fertilizer yields in addition to improving larval performance.

Here, we report for the first-time room and substrate temperature patterns during BSF facilitated composting. Most studies using BSF for waste degradation and composting have focused on monitoring the room temperature only [5,11,58]. The mesophilic temperature (45 °C and below) patterns observed in current studies during the composting process are within the range as reported in previous studies [59]. The highest temperature values observed during the initial stages of the composting process might be attributed to the rapid breakdown of available simple protein and carbohydrate sources for energy by BSF larvae and associated micro decomposers [17]. The mesophilic temperatures are crucial in reducing nitrogen volatilisation during composting [21].

There was a significantly high EC in substrates treated with gypsum during the experiments. This is in line with previous studies where the inclusion of salts of calcium, potassium and aluminium resulted in significant increase in EC values in composts [28,60,61]. However, the EC values of mature compost for substrates amended with gypsum were also within the recommended range (< 4 mS cm^-1^) for field application [45].

This study revealed that biochar amendment of substrates did not influence the compost maturity period as indicated by the high cabbage seed germination rates (> 90%) and germination index values (> 80%) in mature compost extracts for all the treatments. This implies that mature compost from substrates amended with biochar were free of any phytotoxic substances [47]. The low seed germination rates and germination indices observed for compost generated from substrate amended with 5 and 15% gypsum implies moderate phytotoxicity, which might be probably attributed to the high EC. Moderate phytotoxicity of mature compost has been reported to affect seed germination and radical elongation [47]. This study demonstrated that the composting time of substrates amended with gypsum and biochar can be drastically reduced to 5 weeks using BSF larvae for initial breakdown compared to the conventional composting process of 8–24 weeks [17,32,34,59].

A remarkable increase in N retention was observed in frass generated from substrates amended with biochar and gypsum. For gypsum treated frass, N retention could have been influenced by the lower pH values, given that pH levels of 7.5 and below do not favour formation of ammonia gas [21,22,62] but chemically combines to form a more stable ammonium sulphate [28]. Nitrogen conservation in biochar amended frass can be attributed to both adsorption and absorption mechanisms of ammonium ions [23]. For example, higher ammonium retention has been reported for poultry litter amended with biochar [33], which might have been due to inhibition of enzymatic activities of the nitrifying bacteria [33]. However, frass generated from substrate amended with 20% biochar had the lowest N and P concentrations, which could be partially attributed to the high bio-conversion efficiency and nitrogen uptake by larvae reared on this substrate [1,8].

The considerable variation in the trend of K concentration observed throughout the experiments is consistent with previous studies using chicken, pig, and cow manure as rearing substrates for BSF larvae [9,11,58]. However, higher K concentrations were achieved at 20% inclusion of biochar, which can be attributed to the initial higher concentrations of K introduced through biochar amendment [29]. Therefore, biochar amendment is also effective for production of potassium-rich organic fertilizer.

The results of this study indicate that the N concentration in frass fertilizer generated from all amended substrates were within the recommended standards of > 1% N according to the Kenya Bureau of Standards (KEBS) guidelines for optimal commercial organic fertilizer [63]. Also, the nutrient concentrations in the frass fertilizer and level of compost maturity achieved meet the required international standards and guidelines for compost quality [64]. The concentrations of P and K in frass fertilizer are comparable to previous studies by Liu et al. [58], Gao et al. [65] and Oonincx et al. [9].

## Conclusions

The findings of this study have demonstrated for the first time that BSF larvae are capable of efficiently converting substrates amended with up to 20% biochar into high-quality biomass for animal feeds and nutrient rich frass fertilizer for organic farming. The initial composting of biochar amended substrates using BSF larvae significantly shortened the compost maturity time to 5 weeks compared to the conventional 8–24 weeks. Over 90% germination indices achieved for biochar amended frass fertilizer is an indicator of mature and stable organic fertilizer. Therefore, waste composting assisted by BSF should be recommended as a sustainable method of dealing with organic municipal waste that embraces the concept of a circular economy. Being a financially more attractive option for municipal waste management, private sector players, with stronger focus on business and marketing should be in the centre of attention for rapid adoption of this two-pronged approach insect-based technology. This will create new economic opportunities for municipalities and offer small entrepreneurs the possibility of income generation without high investment costs, and concurrently reduce the environmental impact of organic waste streams currently considered as one of the most serious environmental problems confronting urban governments in low- and middle-income countries.
